# Supplementary material for: Rapid turnover of life-cycle-related genes in the brown algae
Source: Genome Biol. 2019 Feb 14;20:35. doi: 10.1186/s13059-019-1630-6 (PMC6374913; doi:10.1186/s13059-019-1630-6)
Supplement: Supplementary file 3 — Protein alignments, corresponding Gblocks html file and CDS sequences for the lineage conserved gametophyte-biased genes. (ZIP 2109 kb) [file 13059_2019_1630_MOESM3_ESM.zip › Con_GA_alignments/Ec-14_004190.aa_ali.fasta-gb.htm]

Ec-14\_004190.aa.fasta.trans.aa\_ali.fasta


## Gblocks 0.91b Results

Processed file: **Ec-14\_004190.aa.fasta.trans.aa\_ali.fasta**  
Number of sequences: **2**  
Alignment assumed to be: **Protein**  
New number of positions: **271** (selected positions are underlined in blue)

```
                         10        20        30        40        50        60
                 =========+=========+=========+=========+=========+=========+
Ec-14_004190     -MISSCWTPDRNELVGKLVRRFAFDFDAVSNAFRNHLQREGVDFKSEETAPAALRKVYAS
MSTRG.18265.1    MTTQFCWTSERQELVGKLVRRFAFDFDAVSNAFRDHLTSEGVDFSPNAMNPRSLRKVFAA
                      #######################################################


                         70        80        90       100       110       120
                 =========+=========+=========+=========+=========+=========+
Ec-14_004190     LDRKRPPRAVESAPLPGVRKENDEPGGEADRTLVDVSGGDWDSMGTGSQ---GKLNGYIM
MSTRG.18265.1    LDRKRAAPAGESSANS-YDQGLGDTDAEGGCGLGDVSYGGWGSSAAKRASREQHADNPIQ
                 ############               ################               ##


                        130       140       150       160       170       180
                 =========+=========+=========+=========+=========+=========+
Ec-14_004190     GNLHSSRSGSVTGEGYNNLPPPERSDQLGASDDCPSEREDVQHTSGSDEGEKSVTAPSSL
MSTRG.18265.1    GALCTHDPGLTARGESKGSSFPGLVVQRDFREGC------VSKESGRNEGTVEATAP---
                 #########            #############      #################   


                        190       200       210       220       230       240
                 =========+=========+=========+=========+=========+=========+
Ec-14_004190     PSTDESGWDFGRFLEEQRLKEIQHDDRKEKIF----------------------------
MSTRG.18265.1    PATDKS-WSFDNFVEDQRRKEIQHDDQKEKLFLKVLSALGGVADGPGPTAELPPDILRAW
                        #########################                            


                        250       260       270       280       290       300
                 =========+=========+=========+=========+=========+=========+
Ec-14_004190     ------------------QK--EDRHLACQRQSLKKRYLLDSEDFIGEDPLGLLDD---T
MSTRG.18265.1    GERNDKLVAKAEEANRRRLKVEEDRKLALQRQSLKQRHLPDSVDCVGDDPLAVLTTGCTA
                                       ################################      


                        310       320       330       340       350       360
                 =========+=========+=========+=========+=========+=========+
Ec-14_004190     TAPAFQQEEGDIAGGLSPR-QSHRQEEAEERLQGDDAHTS-----HQAAAGPVRLELGMD
MSTRG.18265.1    TMPAPEDEEG--SGTLLPRCHPCCREEPEENAGGDDAGASSPRCGGPGVAGPVRLELGMD
                 ##########               ###############         ###########


                        370       380       390       400       410
                 =========+=========+=========+=========+=========+======
Ec-14_004190     AEELDKILNELERESSLAGGGWAETNSELSRVLDELDAAASEPPANAVLLQDTRE-
MSTRG.18265.1    AEVLEKILDEHEREFSLAAGGYAETDSELSRVLDELDEAAARGGNPARLLCNVRDQ
                 ######################################################
```

```
Parameters used
Minimum Number Of Sequences For A Conserved Position: 2
Minimum Number Of Sequences For A Flanking Position: 2
Maximum Number Of Contiguous Nonconserved Positions: 8
Minimum Length Of A Block: 10
Allowed Gap Positions: None
Use Similarity Matrices: Yes
```

```
Flank positions of the 10 selected block(s)
Flanks: [6  72]  [88  103]  [119  129]  [142  154]  [161  177]  [188  212]  [263  294]  [301  310]  [326  340]  [350  414]  

New number of positions in Ec-14_004190.aa.fasta.trans.aa_ali.fasta-gb:  271  (65% of the original 416 positions)
```
